# Sensory Attributes and Instrumental Chemical Parameters of Commercial Spanish Cured Ewes’ Milk Cheeses: Insights into Cheese Quality Figures

**DOI:** 10.3390/foods13010127

**Published:** 2023-12-29

**Authors:** Ana Beltrán Sanahuja, Rafaela Pesci de Almeida, Kilian-Anja Igler Marí, Marina Cano Lamadrid, Arantzazu Valdés García, Esther Sendra Nadal

**Affiliations:** 1 Department of Analytical Chemistry, Nutrition and Food Sciences, P.O. Box 99, 03080 Alicante, Spain; ana.beltran@ua.es (A.B.S.); rafaela.pesci@gmail.com (R.P.d.A.); 2 Centro de Investigación e Innovación Agroalimentaria y Agroambiental (CIAGRO-UMH), Miguel Hernández University, Carretera de Beniel, km 3.2, Orihuela, 03312 Alicante, Spain; kilian-anja.igler@umh.es (K.-A.I.M.); marina.canol@umh.es (M.C.L.); esther.sendra@umh.es (E.S.N.)

**Keywords:** ewe, SPME, protected designation of origin, cheese, sensory analysis

## Abstract

The external appearance of some of the Protected Designation of Origin (PDO) cured cheeses is similar to other cheese samples made in Spain: 1 kg and 2.5–3 kg formats, cylindrical, and with or without a pleita mark on the surface. In this work, commercial cured ewe’s milk cheese samples with a similar external appearance were analyzed, including five PDO and five non-PDO samples. The parameters analyzed were color, texture, pH, humidity, water activity, and the volatile profile. Additionally, a descriptive and consumer-sensory analysis of the cheese samples was carried out. Statistical analysis of the results showed that luminosity, color coordinates a* and b*, percentage of deformation, humidity, water activity, and acid contents were significantly higher in non-PDO cheese samples. The breaking force, maximum force, and the content of esters were significantly higher in those cheese samples with PDO. In addition, PDO cheese samples showed higher scores for all attributes evaluated by consumers, except for color. These results suggest that PDO cheeses are placed on the market with a higher degree of ripening than non-PDO ones and that consequently they are more positively valued by consumers.

## 1. Introduction

The worldwide production of ewe cheese showed an important increase from 645,540 to 683,493 tons between 2010 and 2020 [1]. Nowadays, the production of pure ewe cheese in the EU was around 385,444 tons, with Greece being the leading producer (40%) followed by Italy (19%), Spain (18%) and France (16%) [2]. Ewe cheese is characterized by a high moisture content (38–43%; *p* ≤ 0.05) and a lower percentage of crude protein (23–32%; *p* ≤ 0.01;),in addition to containing a number of total solids around 60% and a value of crude fat ranging from 19 to 29% [3,4,5].

Products with differentiated characteristics due to the origin of their raw materials and/or manufacturing procedures are regulated by European Union Regulation (EC) No. 1151/2012, complemented by Spanish Law 6/201 [6]. If these products meet the conditions established by each specific regulation, they may voluntarily adhere to one of the following certifications: protected geographical indication (PGI), protected designation of origin (PDO) and guaranteed traditional specialties (GTS). These three protection schemes favour recognition of the quality and value of the product, mainly by the consumer, and try to combat the unfair competition to which products of a certain reputation are subjected, since the use of protected names for non-protected products not only affects the producer but is considered food fraud for deceiving the consumer [7]. Also, these designations constitute a structural tool in the development and sustainability of rural traditions, as well as a way of promoting Spanish products abroad [8]. In this sense, it is reported that PDO cheese samples are sold at higher prices than non-PDO ones [9].

The PDO is a European certification of quality that covers different products whose characteristics and quality are based on the place where they are produced or processed [10]. Some of the products covered by the PDO are cheeses, with a total of 26 different cheeses with PDO in Spain. The PDO Regulatory Council allows the production of cheeses in an artisanal way with raw milk and industrially with pasteurized milk. The term “ripened cheese” refers to one that, after the manufacturing process, needs to be kept for a certain time at a suitable temperature and in such conditions that characteristic physical and chemical changes take place. The minimum maturation period is established as 60 days, except for those cheeses made with pasteurized milk and that have a weight ≤ 1.5 kg, for which the minimum maturation is fixed as 30 days with a maximum period of 2 years [11,12,13,14].

The external appearance of PDO cheeses is like other cured ewe cheeses without PDO. However, the final acceptance by consumers depends upon several factors of which flavor is one of the most important [15]. This complex attribute depends upon volatile and non-volatile chemical compounds being released from the main fractions of milk during the ripening process: fat, protein, and carbohydrates. Therefore, each product has a characteristic and unique flavor due to its inherent volatile components [16]. Thus, this work is focused on the comparative study of instrumental quality parameters (color, texture, pH, water activity, and the volatile-compounds profile) between ewe’s milk cheeses with or without PDO. In addition, a descriptive sensory analysis and an affective consumer’s study have been carried out to compare the PDO and the non-PDO cheese samples. As a result, the development of fast, sustainable, and reliable techniques for the direct analysis of samples in combination with multivariate analyses is proposed as a useful tool to investigate PDO vs non-PDO cured cheese samples made with ewe’s milk, and for the control of cheese samples during food processing to ensure their authenticity.

## 2. Materials and Methods

### 2.1. Cheese Samples

To select the cheese samples for analysis brands with the highest consumption in kg, thus considered the most popular brands, were sampled. That is, the brands sold in the most popular supermarket chains in Spain. The selected cheese samples are all industrially made. Three whole pieces from different batches of those same brands were purchased in single or multiple establishments of those supermarket chains. In this way, differences due to individual pieces of cheese were avoided. So, the studied samples were 30 pieces of cured ewe’s milk cheese (3 different batches from 10 different samples) purchased in supermarkets according to the availability offered (Table 1). The following sample inclusion criteria were used: cheese made with pasteurized or raw ewe’s milk, minimum maturation time of 30 days, and pieces weighing 3 kg. All the cheese pieces were stored under refrigerated conditions by using vacuum packaging until the day before the analysis.

### 2.2. Sample Preparation

Moisture, volatile compounds, pH, and water activity analyses were run using ground cheese samples. For texture and color analyses, a piece comprising 1/3 of the cheese cylinder was used. Slices 2 cm-thick were obtained after discarding 1.5 cm from the rind. The color was measured from the slices’ surface, and immediately afterward the slices were used to obtain cylinders for texture analysis [17,18]. For sensory analysis, triangle shape slices were provided to panelists.

### 2.3. Optimization of HS-SPME Procedure

Although HS-SPME analysis of volatile compounds by from ewes’ cheeses, such as Manchego, has been previously described, the optimization of this procedure by using a Box–Behnken design (BBD) has not been yet reported [19,20]. In this study, three independent factors were considered: extraction temperature (A = 35, 52.5, 70 °C), extraction time (B = 15, 37.5, 60 min), and the amount of sample (C = 0.5, 1.75, 3 g). A total of 17 experiments (3-level design, including 5 center points) were carried out in a randomized order. The sample used for the optimization was a sample of cured ewe’s cheese with PDO. The response variable studied was the sum of the total areas of all the volatile compounds extracted, as it was described previously (Table 2).

The design includes the performance of regression analysis of the experimental data so that they fit a second-order polynomial mathematical model described with the following empirical Equation (1):

Y = β_0_ + ∑ β_i_X_i_ + ∑ β_ii_X_i_ + ∑ β_ij_X_i_X_j_ (1)

where Y is the predicted response; X represents the system variables; i and j are the design variables; β_0_ is a constant; β_i_ is the linear coefficient; β_ii_ is the quadratic coefficient; and β_ij_ is the coefficient of the interactions between variables.

### 2.4. Analysis of Texture, Color, pH, Humidity, and Water Activity

The color was analyzed by using a previously calibrated CM-2600d KONICA MINOLTA (Tokyo, Japan) color spectrophotometer, taking 18 random measurements on freshly created surfaces of the cheese, with nine measurements belonging to the outer zone and nine to the interior area. Measurements were taken of the luminosity and the a* and b* color coordinates of the CIELab color space.

Cylinders with 1 cm diameter were obtained from the 2 cm-thick slices with a 1 cm diameter punch. In total, 18 cylinders were obtained, nine belonging to the outer zone and nine from the inner zone. All of them were held at 7 °C for 4 h before performance of the uniaxial compression test. The compression of cubes was achieved to 80% of their original height. All tests were carried out at a constant temperature of 7 °C. The texture analyzer used in this study was the TA-TX2 model (Stable Micro System Ltd., Surrey, United Kingdom) with a 245 N load cell and a crosshead speed of 50 mm min^−1^. Fracture strength, deformation at fracture, work for fracture, and the maximum force for compression were calculated.

The pH determination was carried out by means of a potentiometric analysis with a Basic 20 Crison Instruments S.A (Barcelona, Spain) model digital pH-meter, using the method proposed by [21]. Five grams of cheese was mixed under stirring with 50 mL of distilled water for 5 min to homogenize the sample, and measurements were later taken with the potentiometer electrode. Samples were measured in triplicate.

Moisture was determined by using the method proposed by [21]. For this purpose, five grams of cheese were weighed and placed in a JP Selecta Conterm model oven (Barcelona, Spain) for 24 h at 105 °C. After this time had elapsed, the sample was weighed again and the difference in weight was considered the water content of the sample. Samples were measured in triplicate.

The water activity was measured using a previously calibrated AwSprint TH-500 Novasina AG Labware (Lachen, Switzerland) instrument. Cheese samples (grated) were placed in the hygrometer and water activity was measured at 25 °C. Samples were analyzed in duplicate.

### 2.5. Sensory Analysis

#### 2.5.1. Descriptive Sensory Analysis

Ten highly trained panelists (aged 30 to 55 years) with more than 600 h of training in sensory testing from the Department of Agro-Food Technology (UMH) participated in this study (Food Quality and Safety Group). Also, a member of the University of Alicante (Analytical Chemistry, Nutrition and Food Sciences Department) was presented in the two different sessions in which cheeses were evaluated. The selection and training of the panel followed the ISO standard 8586-1 [22]. For the present study, the panel worked during 1 orientation session (90 min) during which they trained and discussed the main organoleptic characteristics to be used in the final evaluation. The cheese lexicon used in this session was previously described by other authors [23,24,25,26,27]. A unipolar and numerical scale was used to obtain the sensorial information of panelists in which 0 was no intensity and 10 expressed an extremely high intensity. Punctuation of the scale used an increment of 0.5 [28,29,30,31]. All samples were presented with 3-digit codes and randomized numbers. To cleanse the sensations of cheese samples, unsalted crackers and water were provided between samples.

#### 2.5.2. Affective Sensory Analysis

Consumers (*n* = 100) were recruited among the students/professors of the Food Science and Technology/Agro-Food Engineering Degree at the Miguel Hernández University (UMH, Alicante, Spain) and the Gastronomy Degree at the Alicante University (UA, Alicante, Spain). Their ages ranged from 18 to >45 years old (42%: 18–24 years old; 24%: 25–35 years old; 12%: 36–45 years old; and 22%: >45 years old), being 61% females and 39% males. More than 75% of the consumers were regular consumers of sheep’s milk cheese and regular household buyers. An affective consumer’s test was carried out following a balanced design of incomplete blocks (split-plot). Authors needed to conduct a study to evaluate 10 products but for this type of evaluation and product type, consumers can give reliable ratings only if 5 products or less are evaluated. Therefore, the described design was selected and, in this way, all of the samples were analyzed by the same number of panelists, but not all the panelists evaluated all the 10 samples. This design was carried out with the support of XLSTAT software [32]. The overall satisfaction degree using a 9-point hedonic scale, where 1 meant “dislike extremely” and 9 meant “like extremely”, with a “neither like point” denoted 5. Also, questions about the intensity of the attributes using a JAR (just about right) scale were presented. The attributes analyzed were global satisfaction, color, smell, hardness, crumbliness, acidity, saltiness, spiciness, ewe flavor, and aftertaste.

### 2.6. Statistical Analysis

StatGraphics Centurion XV software (version 5.1) was used for the optimization of the HS-SPME procedure (Statistical Graphics Corporation. Rockville, FL, USA). XLSTAT (Microsoft Corporation, Redmond, WA, USA) and StatGraphics Plus 5.0 softwares (Manugistics Inc., Rockville, FL, USA) were used for analyzing the data obtained from the sensory analyses. Statistical tests were performed for the obtained results using the SPSS program with a *p*-value < 0.05. One-factor ANOVA with a Tukey po-t hoc test were carried out to identify statistically significant differences among the studied samples.

## 3. Results

### 3.1. Optimization of Volatiles Extracted from Hard-Cured Ewe Cheese Samples by HS-SPME

#### 3.1.1. Fitting the Model

Table 2 shows the BBD and the responses based on the sum of total areas of all the volatile compounds extracted from the samples of cured ewe’s cheese with PDO. Equation (2) details the polynomial equation of second order, in which the studied response (total area) is expressed as a function of the independent variables (A: extraction temperature; B: extraction time; C: sample weight):

Response = −8.05 × 10^9^ + 2.42 × 10^8^ × A + 1.42 × 10^8^ × B − 3.89 × 10^8^ × C − 1.67 × 10^6^ × A^2^ − 939,482 × AB + 1.46 × 10^7^ × AC − 1.28 × 10^6^ × B^2^ + 8.45 × 10^6^ × BC − 1.03 × 10^8^ × C^2^ (2)

Table 3 shows the ANOVA analysis that was carried out to study the reliability of the fitted model. The good precision of the model is shown by the high *p*-value (0.6067) that indicates no significant ability of the lack-of-fit or the goodness-of-fit of the model to predict the studied response. Also, the R^2^ coefficient of the quadratic regression model was 0.7480.

#### 3.1.2. Effects of Independent Variables on Volatiles Extraction

**Figure 1:**
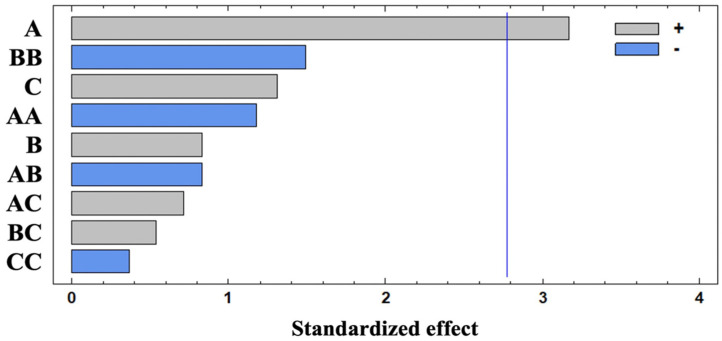
Pareto chart obtained for total sum of areas of volatiles compounds extracted by HS-SPME. The vertical line indicates the statistical significance at 5% of the effects.

Figure 1 shows that volatiles extraction was only significantly influenced (95% confidence) by the positive effect of the extraction temperature (A). Thus, the extraction yield increased with temperature, and was proportional to the increase in the analyte diffusion coefficient. Bezerra et al. reported similar results when optimization of the volatile-compounds extraction in caprine Coalho cheese was carried out using a response-surface methodology [33]. However, a negative A2 interaction effect has been shown. This fact could be related to the partial degradation of some compounds linked to the formation of aromatic compounds due to the Maillard or caramelization reactions.

No significant impact on the studied response (*p* > 0.05) was observed for the rest of the studied parameters. The influence and positive (+) or negative (−) effects of these variables on the response were in the following order: B2 (−) > C (+) > A2 (−) > B (+) > AB (−) > AC (+) > BC (+) > C2 (−). In this sense, individual effects of extraction time (B) and sample weight (C) were positive whereas their quadratic effects (B2 and C2) were negative because of the saturation of the closed vial headspace and, subsequently, the fiber coating [34]. Finally, AC and BC interactions were positive, suggesting that a higher sample weight requires higher extraction temperatures and time. However, the negative effect of the AB interaction could promote the degradation of the volatile compounds [35].

#### 3.1.3. Determination of Optimal HS-SPME Conditions

The optimal extracting conditions obtained in the present study were a temperature if 70 °C, a 40 min extraction time, and a 3.0 g sample weight. Under these conditions, the response value predicted by the model was 3.7 × 10^9^. To ensure the reliability of the proposed model, verification experiments under the optimal conditions were performed, in triplicate, obtaining an experimental value of 2.9 × 10^9^ ± 0.3 × 10^9^, which did not differ significantly from the predicted value by the model. In conclusion, the developed quadratic model was reliable for the HS-SPME optimization of volatile compounds from hard-cured ewe cheeses.

### 3.2. Characterization of the Main Volatile Compounds in Spanish Hard-Cured Ewe Cheeses Using HS-SPME–GC–MS

**Figure 2:**
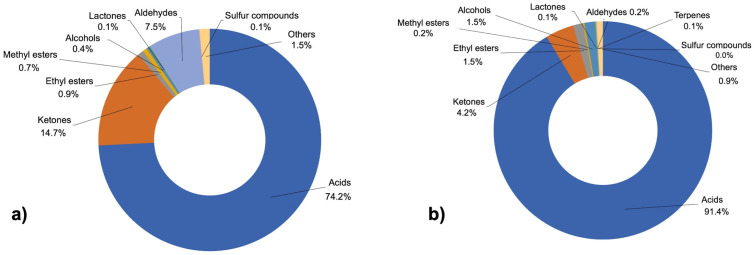
Average percentages of the classes of volatile compounds found in PDO (**a**) and non-PDO (**b**) cheese samples.

Firstly, a qualitative study was carried out based on the sum of relative areas to explore differences between samples with and without PDO. More than 55 compounds were identified from the studied samples. Figure 2 shows the average percentage of the classes of volatile compounds found in the cheeses. In general, PDO samples (Figure 2a) were characterized by organic acids (74.2%) followed by ketones (14.7%), aldehydes (7.5%), ethyl (0.9%) and methyl esters (0.7%), and other compounds. In contrast, samples without PDO (Figure 2b) were characterized by a high content of organic acids (91.2%), ketones (4.2%), alcohols (1.5%), ethyl esters (1.5%), and other compounds. The most common compounds detected in the samples are detailed in Table 3 and have been previously detected in various cured ewes’ cheeses based on previous studies [36,37,38].

The ripening process of cheese is very complex and involves microbiological and biochemical changes to the curd that result in the characteristic flavor and texture of each variety. Biochemical changes in cheese samples during ripening may be grouped into primary events (lipolysis; proteolysis; and metabolism of residual lactose, lactate, and citrate) or secondary events (metabolism of fatty acids and of amino acids) [39]. Enzymes with lipolytic activity (esterases and lipases) may cause the release of linear-chain acids (hexanoic, nonanoic, octanoic, decanoic and dodecanoic acids) [15]. All of them are related to the fatty, cheesy, and waxy flavors of samples. The abundant presence of these short-chain linear fatty acids is thought to be responsible for the characteristic piquant taste of ewes’ cheeses [40]. Carboxylic acids are also precursors of other aromatic compounds, such as ketones, lactones, aldehydes, and esters [41,42]. All of them were extracted and identified in the present work. Consequently, it is expected that PDO samples show a higher ripening stage, due to the high content on secondary aromatic metabolites, in contrast to the non-PDO samples, which are mainly characterized by carboxylic acids.

Using the optimized HS-SPME–GC–MS procedure, 14 volatile compounds were selected for this study based on two criteria. On one hand, the volatile compound had to be present in all the studied samples. On the other hand, identification comprising a minimum similarity of 90% with a compound present in the NIST database was mandatory. Their relative abundance was determined within the selected cheese samples. Identified compounds (Table 4) were grouped under six headings: five carboxylic acids, three esters, three ketones, one aldehyde, one lactone, and one oxime.

Three main esters (ethyl hexanoate, ethyl octanoate and ethyl decanoate) were identified in the samples. The presence of esters in cheese samples is related to the esterification of free fatty acids with alcohols both by chemical and enzymatic reactions [44]. Ethyl esters are especially known for their important role in the formation of a fruity character in cheese. These compounds can contribute to the aroma of cheese by minimizing the sharpness and the bitterness imparted by fatty acids and amines, respectively [44]. The most abundant ester in the studied samples was ethyl decanoate which has been previously described for cured ewes’ cheeses [45]. The ethyl hexanoate is considered a key odorant in some cheese varieties such as blue cheeses and grana-type cheeses [40] and gives a ‘fruity’ off-flavor in Cheddar [46], whereas ethyl octanoate seems to be significant in the formation of the aroma of Flor de Guia cheese [44].

Ketones are formed by the enzymatic oxidation of fatty acids to keto-acids and their consequent decarboxylation to methyl ketones [21]. In the studied samples, the identified ketones were 2-heptanone, 2-nonanone, and 2-undecanone. 2-heptanone was the most abundant ketone detected in this work, in accordance with the literature, which highlighted that this ketone could play an important role in the final fruity, floral, and musty aroma notes of cheeses made from raw ewes’ milk such as Zamorano, Manchego and La Serena [19,45]. Fruity, floral, and musty notes are associated with 2-nonanone and 2-undecanone, whereas blue cheese notes are attributed to 2-heptanone [44].

Only one aldehyde (phenylacetaldehyde) was identified in all of the studied samples. In effect, aldehydes are transitory compounds that do not accumulate in cheese because they are produced from amino acids either by transamination followed by decarboxylation or by Strecker degradation, being quickly transformed into alcohols or the corresponding acids [44,45]. Therefore, a low level of aldehydes indicates a good ripening of the cheese, whereas a high concentration of aldehydes may cause off flavors [42]. The aromatic aldehyde phenylacetaldehyde is formed by phenylalanine degradation, via a Strecker reaction [47]. This compound has been reported in the literature as the main benzenic compound quantified in Spanish cured ewes’ cheeses and has been related to honey-like and floral aromas [44,45].

Only one lactone was detected in all the studied samples, δ-decalactone, which has been found in the highest concentrations in cured ewes’ cheeses [45]. Lactones have fruity, sweet, creamy, and fermented notes [15]. In particular, δ-decalactone contributes to the pleasant coconut odor note [44]. δ-Lactones have been reported that may be formed from δ-hydroxyacids following their release from triacylglycerides by lipolysis. So, concentration of lactones usually correlates with lipolysis extent [48].

Finally, there is little information on methoxyphenyl oxime, but the compound has been found in pasteurized cows’ milk [49]. In a different study, cheeses made with cows’ milk with the *Streptococcus thermophilus* microorganism had a higher content of methoxyphenyl oxime than other cheeses, whereas there was an absence of this compound in directly acidified cheeses, suggesting that it might be produced by bacteria [50].

**Figure 3:**
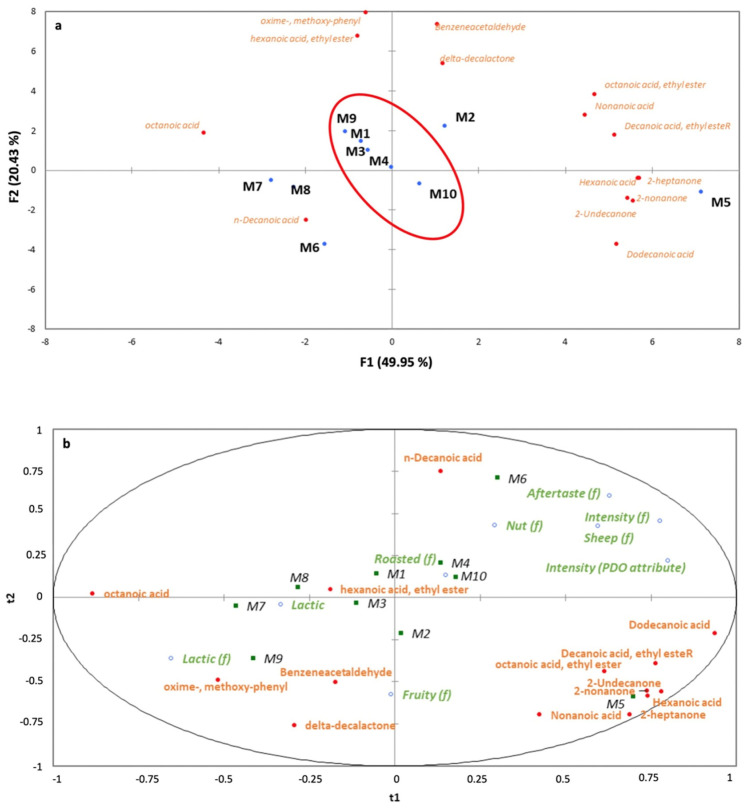
Multivariate analysis: (**a**) Plot of the results from Principal Component Analysis performed using the results of the detected volatile compounds; (**b**) Plot of the PLS obtained by using the results of the relationships between volatiles and odor and flavor descriptors.

Based on ANOVA and the Tukey post hoc test (Table 4), it can be confirmed that the volatile profile differs significantly when comparing the studied samples. The variability for all volatiles obtained by Tukey analysis highlighted the necessity of carrying out a multidisciplinary statistical approach in this study. The PCA applied to the 14 volatiles showed two main principal components accounting for 70.37% of the total variation (49.95% F1, 20.43% F2). Projections of sample scores on the plot are shown in Figure 3a. The first function clearly showed differences in the plot space among the samples. In fact, samples with PDO (M1, M3, M4, M9, and M10) were placed closer to each other, showing a homogeneous group in contrast to non-PDO samples (M2, M5, M6, M7, and M8) which were more dispersed in the space.

Taking into account the average values of the identified six chemical families, PDO samples were characterized as having 93.80% composition by acids, 3.72% by oxime compounds, 1.01% by alkyl esters, 0.83% by ketones, 0.46% by lactone and 0.18% by aldehyde compounds, whereas the non-PDO samples showed higher content of acids (94.66%) followed by oxime (2.47%) and ketones (1.57%), with lower contents of esters, lactone, and aldehyde with 0.77%, 0.39% and 0.13%, respectively. All the chemical families showed statistically significant differences when comparing PDO and non-PDO samples (*p* < 0.05), except for ketones and methoxyphenyl oxime compound. Since carboxylic acids are precursors of other aromatic compounds, such as ketones, lactones, aldehydes, and esters [42], these results seem to indicate that the cheeses with PDO show a more advanced maturation stage, with lower content of carboxylic acids but higher contents of esters, phenylacetaldehyde, and δ-decalactone than the non-PDO samples. This finding agrees with those reported for volatile profiles of Spanish cured cheese with the PDO “Manchego”. In this work, samples were studied in two different stages of ripening (4 and 8 months) [51]. In conclusion, secondary metabolites such as ketones and esters increased their concentrations during the ripening time.

### 3.3. Color, Texture, Humidity, Water Activity and pH Analysis

Texture can be defined as an attribute of cheese originating from a group of physical properties [52]. The texture is determined from the breaking force (force needed to break the cylinder, the higher the force the harder the cheese), the deformation at the breaking point (the higher the deformation the more elastic), the breaking work, and the maximum force exerted on the cheeses. A significantly higher mean percentage of deformation of the non-PDO cured cheeses (*p* < 0.001) was observed, whereas the mean breaking force and the maximum force are significantly higher (*p* < 0.001) in the cheeses with PDO. In terms of the breaking work, no significant differences were observed between the cured cheeses with or without PDO (Table 5). The obtained results are probably related to the duration of the curing process, since as the maturation advances, the cheese becomes more rigid and less elastic, as has been reported previously by Picon et al. for Manchego cheese [53] and Colin et al. for Cheddar samples in which increased age resulted in increased hardness [54]. So, the texture results indicated that the more cured cheeses would be those with the PDO. Texture results were in accordance with those for volatiles, as PDO samples showed lower contents of carboxylic acids but higher contents of esters, phenylacetaldehyde, and δ-decalactone contents than the non-PDO samples.

Maturity of cheese is a complicated process that involves several concurrent and interlinked reactions, including pH changes, lipolysis, and proteolysis [55]. Cheese color is also an important physical parameter, which can be considered as an indicator for many attributes like flavor, microbiological quality, and maturity [56]. The average color properties of the cheeses, measured using the CIELab color space, show that lightness (L) and the a* and b* coordinates present significantly higher values (*p* < 0.001) in non-PDO cheeses (Table 5). These differences in color may also be associated with the curing time of the cheese samples, as color is a good indicator of their maturity. The most cured cheeses seem to be those with PDO, as their colors are more yellow-brown as previously described [57].

On the other hand, no significant differences have been detected in the pH values of the analyzed cheese samples whereas the average obtained values of moisture and water activity are significantly higher (*p* < 0.001) in non-PDO cheese samples as is shown in Table 5. A possible more advanced maturation stage of the cheese with PDO sampled also explains the results as there is a more pronounced loss of water in these samples [58,59].

Therefore, maturation can be considered a process by which the cheese loses water, becoming harder and taking on a stronger flavor. Based on the obtained results, the cheese samples with PDO show, on average, lower values of the color parameters, humidity, and lower water activity, as well as a lower percentage of deformity, but they present a higher breaking force and maximum force. These results seem to indicate that the cheeses with PDO present a more advanced maturation stage than the cured ewe cheeses that do not have the PDO, which is in keeping with volatile compounds results. 

### 3.4. Descriptive Sensory Analysis

Thirty attributes showed significant differences among all studied samples. There were statistically significant differences for five visual descriptors (external and internal color, brightness, color homogeneity, and the presence of eyes), six olfactive descriptors (overall intensity PDO ID, lactic, sheep, roasted, fruity (pineapple), and butter), six textural descriptors (touch, firmness, elasticity, crumbliness, graininess, and creaminess), five basic taste descriptors (sourness, saltiness, umami, sweetness, and bitterness), and eight flavor descriptors (intensity, lactic, sheep, roasted, fruity, aftertaste, nutty, and spicy). A partial least squares plot (PLS) was shown to allow a better understanding of the relationships among the ten cured ewes’ cheeses, using (i) volatile compounds and (ii) descriptive sensory attributes (Figure 3b). PDO samples (M1, M3, M4, M9, and M10) that can be associated with lactic odor and three flavors (lactic, roasted, and fruity) that can be associated with the three main volatiles situated in the plot near the samples (ethyl hexanoate, phenylacetaldehyde, and δ-decalactone). As was previously described, these compounds are reported in the literature as possible markers of a more advanced maturation stage. Additionally, in Figure 3a, non-PDO samples (M2, M5, M6, M7, and M8) were more dispersed in the space, showing more heterogeneity within this group.

### 3.5. Affective Sensory Analysis

Two PDO samples showed the highest overall similarity values (M3 and M4) whereas the M7 (non-PDO) showed the lowest value (Table 6). M3 also showed the highest value for color with the lowest values shown by M5 and M9. Regarding firmness, again the best satisfaction degrees were obtained for M3 and, in this case, M6 and M10, whereas the lowest ones were those of the samples M2 and M8. The highest crumbliness values were those of the samples M5 and M10, with the lowest values being found for the samples M2, M4, M7, M8, and M9. In general, all samples showed similar aftertaste satisfaction degrees, except for M5 and M6, both non-PDO samples, which showed the lowest values.

**Figure 4:**
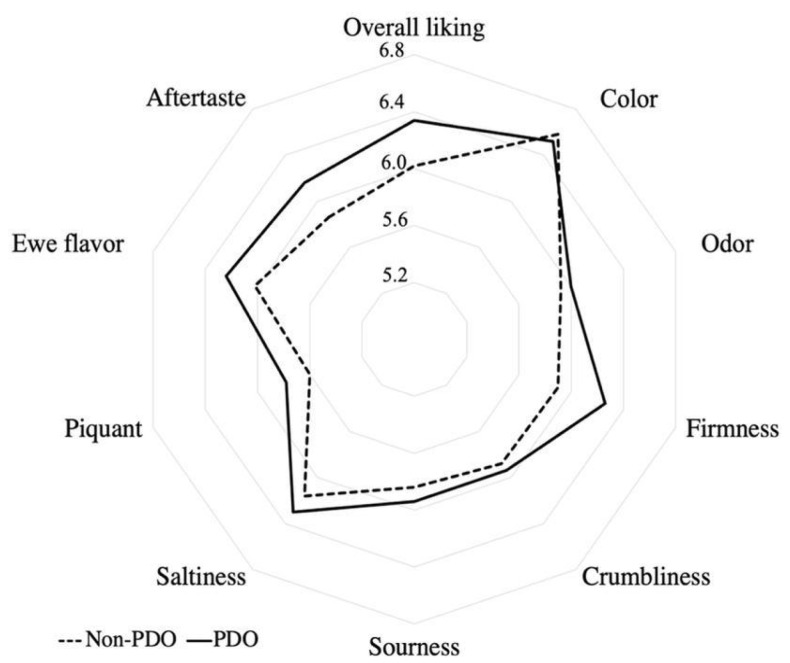
Sensory profiles of PDO and non-PDO Spanish cured ewes’ cheeses obtained by the consumer panel.

Sensory profiles of PDO and non-PDO Spanish cured ewes’ cheeses reported by the consumer panel are plotted in Figure 4. Significant differences (*p* < 0.001) between the PDO and non-PDO groups were found for all attributes studied by consumers (Table 6). In general, PDO cheeses showed higher scores than non-PDO ones, except for the color. As a result, it is noticeable that cheeses covered by the PDO obtained a consumer satisfaction degree higher than non-PDO samples.

## 4. Conclusions

HS-SPME–GC–MS, texture, color, humidity, water activity, pH analysis, and sensory analysis have proved to be valuable and reliable parameters for the characterization of different Spanish ewes’ cheeses. All of them have been shown to be simple and inexpensive techniques suitable to be used for the control of cheese samples in the food processing industry. The techniques used have shown several advantages such as short experimental time, no use of chemical reagents, and requirement of only a small sample size with minimal sample preparation.

The obtained results suggest that PDO samples had a more controlled production procedure compared to non-PDO samples, as the volatile composition allowed the separation of PDO from non-PDO samples, maybe due to a similar ripening stage of samples. Some indicators were found that could suggest that cheeses with PDO are more mature than other ewe’s mature cheeses. For example, lower numbers of carboxylic acids (hexanoic, octanoic, nonanoic, decanoic, and dodecanoic acids) were linked to higher contents of esters (ethyl hexanoate, ethyl octanoate, and ethyl decanoate), δ-decalactone, and phenylacetaldehyde. Also, PDO samples showed higher values for hardness, less water, and more yellowish-brown tones caused by the Maillard reaction, as well as the different textures of these cheese samples that were detected by consumers. In the descriptive analysis, significant differences were observed in 30 attributes. Results obtained from PLS related the PDO samples with lactic, roasted, and fruity flavors, and also with lactic odor, in keeping with the volatile compound results. Attributes analyzed by consumers were better scored for PDO cheeses compared to non-PDO ones. Moreover, the development of the PDO label for Spanish cured ewes’ cheeses, showing they have been manufactured and ripened in a defined geographical area, is a valuable tool to help with the preservation and distinction from other industrially made products.

## Figures and Tables

**Table 1:** Information about the analyzed cured-sheep-cheese samples.

Sample Code	PDO	Minimum Maturation	Origin	Ingredients	Nutritional Information (100 g)
M1	Yes	-	-	Raw Manchega sheep milk, animal rennet, lactic ferments, egg lysozymes, non- edible crust	Energy value 437 kcal, fat 38 g (saturated 34 g), protein 24 g, salt 1.7 g
M3	Yes	-	Ciudad Real	Raw sheep milk, animal rennet, lactic ferments, salt, egg lysozyme, natamycin preservative	Energy value 461 kcal, fat 39 g (saturated 26 g), protein 23 g, salt (g) 1.8 g
M4	Yes	4–5 months	Navarra	Raw sheep milk, rennet, Spanish dairy crops, salt, egg lysozyme, milk and derivatives, eggs	Energy value 478 kcal, fat 41.5 g (saturated 27.3 g), protein 26 g, salt 1.7 g
M9	Yes	4 months	Ciudad Real	Pasteurized La Mancha sheep’s milk, salt, lactic ferments, rennet, E-252, calcium chloride	Energy value 422 kcal, fat 36 g (saturated 23 g), protein 24 g, salt 1.7 g
M10	Yes	6–12 months	Cuenca	Raw sheep’s milk, lactic cultures, calcium chloride, salt, rennet, lysozyme preservative	Energy value 467 kcal, fat 33 g, (saturated 18.7 g), protein 23 g, salt 1.5 g
M2	No	-	Palencia	Pasteurized sheep’s milk, salt, rennet, calcium chloride, E-1105, lactic ferments	Energy value 458 kcal, fat 36 g (saturated 25 g), protein 25 g, salt 1.7 g
M5	No	3–4 months	Cádiz	Pasteurized sheep’s milk, salt, rennet, lactic cultures, egg lysozyme	Energy value 425 kcal, fat 34.04 g (saturated 24.15 g), protein 25.61 g, salt 1.8 g
M6	No	10–14 months	Madrid	Milk, salt, rennet, E-509, egg lysozyme, lactic ferments, E202, natamycin, E172	Energy value 485 kcal, fat 41 g (saturated 28 g), protein 28 g, salt 1.7 g
M7	No	-	Madrid	Pasteurized sheep milk, lactic ferments, salt, rennet, E-252, E-202, E-235	Energy value 441 kcal, fats 36.40 g (saturated 26 g), proteins 27 g, salt 1.6 g
M8	No	-	-	Raw sheep’s milk, rennet, lactic ferments, salt; may contain egg	Energy value 434 kcal, fat 7 g (saturated 26 g), protein 25 g, salt 1.5 g

**Table 2:** Combinations of experimental conditions for the BBD and the measured sum of total areas of all the volatile compounds extracted from a sample of cured ewe’s cheese with PDO.

Run	Factor A	Factor B	Factor C	Sum of Areas
1	52.5	15	3	1.32 × 10^9^
2	70	60	1.75	1.76 × 10^9^
3	70	37.5	3	4.03 × 10^9^
4	35	37.5	0.5	5.56 × 10^8^
5	52.5	15	0.5	1.10 × 10^9^
6	52.5	37.5	1.75	2.18 × 10^9^
7	52.5	37.5	1.75	2.27 × 10^9^
8	70	15	1.75	2.70 × 10^9^
9	52.5	37.5	1.75	2.36 × 10^9^
10	70	37.5	0.5	2.44 × 10^9^
11	52.5	60	3	3.05 × 10^9^
12	52.5	60	0.5	1.88 × 10^9^
13	52.5	37.5	1.75	2.19 × 10^9^
14	35	15	1.75	4.80 × 10^8^
15	52.5	37.5	1.75	4.24 × 10^9^
16	35	60	1.75	1.01 × 10^9^
17	35	37.5	3	8.76 × 10^8^

**Table 3:** Analysis of variance (ANOVA) for the quadratic model of volatiles extraction.

	Sum of Squares	Freedom Degrees	Mean Square	F-Value	*p*-Value
A	8.02 × 10^18^	1	8.02 × 10^18^	10.04	0.0339 *
B	5.49 × 10^17^	1	5.49 × 10^17^	0.69	0.4537
C	1.38 × 10^18^	1	1.38 × 10^18^	1.72	0.2595
AA	1.10 × 10^18^	1	1.10 × 10^18^	1.38	0.3057
AB	5.47 × 10^17^	1	5.47 × 10^17^	0.69	0.4543
AC	4.06 × 10^17^	1	4.06 × 10^17^	0.51	0.5153
BB	1.77 × 10^18^	1	1.77 × 10^18^	2.22	0.2108
BC	2.26 × 10^17^	1	2.26 × 10^17^	0.28	0.6229
CC	1.09 × 10^17^	1	1.09 × 10^17^	0.14	0.7305
Lack-of-fit	1.64 × 10^18^	3	5.47 × 10^17^	0.68	0.6067
Pure error	3.20 × 10^18^	4	7.99 × 10^17^		
Total (corr.)	1.92 × 10^19^	16			

A, B, and C represent extraction temperature, extraction time, and sample amount, respectively. * Significant *p* < 0.05.

**Table 4:** Percentages of peak area of the total volatile composition of cheeses obtained using HS-SPME/GC–MS analysis directly calculated from total ion current (TIC; mean ± standard deviation (SD), *n* = 3), retention times (min), odor (O), and flavor (F) descriptors between PDO and Non-PDO groups.

Code ^b^	Rt	O	F	M1	M2	M3	M4	M5	M6	M7	M8	M9	M10	ANOVA PDO/Non-PDO	*p*-Value
1	15.7	Sour, fatty	Cheesy, fruity	0.03 ± 0.01 ^a^	0.19 ± 0.05 ^c^	0.04 ± 0.01 ^ad^	0.04 ± 0.01 ^ad^	0.60 ± 0.02 ^e^	0.01 ± 0.00 ^a^	0.02 ± 0.00 ^a^	0.04 ± 0.01 ^ad^	0.09 ± 0.01 ^bd^	0.12 ± 0.02 ^b^	***	0.0004
2	18.3	Ni	Ni	5.38 ± 0.98 ^a^	3.36 ± 0.50 ^b^	3.86 ± 0.04 ^c^	2.52 ± 0.42 ^b^	2.52 ± 0.40 ^b^	2.31 ± 0.03 ^b^	2.85 ± 0.27 ^b^	3.31 ± 0.09 ^a^	4.71 ± 0.61 ^a^	2.11 ± 0.21 ^b^	NS	0.4053
3	18.9	Fruity	Fruity	0.23 ± 0.03 ^a^	0.18 ± 0.08 ^ab^	0.12 ± 0.01 ^b^	0.17 ± 0.03 ^ab^	0.02 ± 0.01 ^cd^	0.03 ± 0.01 ^cd^	0.05 ± 0.01 ^cd^	0.06 ± 0.00 ^d^	0.12 ± 0.01 ^bd^	0.13 ± 0.01 ^b^	***	0.0003
4	20.3	Sour, fatty	Sour, fruity	17.00 ± 5.71 ^a^	15.73 ± 2.27 ^a^	14.84 ± 1.72 ^a^	18.61 ± 0.68 ^a^	54.90 ± 3.68 ^b^	7.22 ± 0.18 ^c^	3.77 ± 0.07 ^c^	7.23 ± 0.17 ^c^	7.53 ± 0.12 ^c^	12.34 ± 1.58 ^ac^	NS	0.1952
5	21.8	Green	Honey	0.14 ± 0.02 ^a^	0.36 ± 0.01 ^c^	0.26 ± 0.05 ^d^	0.09 ± 0.01 ^a^	0.12 ± 0.01 ^a^	0.03 ± 0.01 ^b^	0.10 ± 0.01 ^ab^	0.05 ± 0.00 ^b^	0.24 ± 0.01 ^d^	0.16 ± 0.01 ^a^	***	0.0004
6	22.6	Fruity	Cheesy	0.31 ± 0.05 ^a^	0.79 ± 0.20 ^c^	0.10 ± 0.01 ^a^	0.10 ± 0.01 ^a^	4.03 ± 0.37 ^d^	0.06 ± 0.00 ^a^	0.04 ± 0.00 ^a^	0.06 ± 0.01 ^a^	0.30 ±0.03 ^a^	2.14 ± 0.16 ^b^	*	0.0468
7	25.6	Waxy	Waxy	0.34 ± 0.02 ^ac^	0.35 ± 0.07 ^ac^	0.25 ±0.02 ^ad^	0.26 ± 0.05 ^ad^	0.42 ± 0.07 ^c^	0.06 ± 0.01 ^b^	0.02 ± 0.00 ^b^	0.03 ± 0.01 ^b^	0.12 ± 0.01 ^bd^	0.21 ± 0.02 ^d^	***	0.0005
8	26.3	Fatty, cheesy	Soapy, brandy	37.74 ± 1.67 ^a^	37.59 ± 0.92 ^a^	36.40 ± 1.37 ^a^	32.07 ± 3.22 ^ce^	10.57 ± 0.71 ^d^	28.45 ± 1.47 ^e^	82.16 ± 0.27 ^f^	46.42 ± 0.97 ^b^	50.94 ± 2.00 ^b^	34.26 ± 1.90 ^ac^	**	0.0043
9	28.6	Floral	Sweat, cheese	0.17 ± 0.03 ^a^	0.22 ± 0.10 ^a^	0.06 ± 0.01 ^a^	0.04 ± 0.02 ^a^	1.69 ± 0.19 ^c^	0.01 ± 0.00 ^a^	0.04 ± 0.01 ^a^	0.05 ± 0.01 ^a^	0.07 ± 0.01 ^a^	0.56 ± 0.10 ^b^	**	0.0014
10	28.8	Waxy, cheesy	Cheesy, creamy	0.73 ± 0.03 ^a^	1.81 ± 0.15 ^c^	0.82 ± 0.04 ^a^	1.48 ± 0.01 ^b^	2.18 ± 0.25 ^d^	0.75 ± 0.06 ^a^	0.92 ± 0.03 ^a^	0.49 ± 0.03 ^a^	1.58 ± 0.04 ^bc^	0.75 ± 0.10 ^a^	*	0.0217
11	30.9	Sweet, apple	Waxy, fruity	0.54 ± 0.09 ^a^	0.94 ± 0.17 ^b^	0.63 ± 0.04 ^a^	1.00 ± 0.36 ^ab^	1.28 ± 0.21 ^b^	0.32 ± 0.01 ^c^	0.03 ± 0.01 ^d^	0.06 ± 0.01 ^d^	0.33 ± 0.07 ^c^	0.63 ± 0.06 ^a^	**	0.0025
12	31.6	Fatty, citrus	Waxy, fruity	35.39 ± 4.87 ^ac^	35.36 ± 2.81 ^a^	39.80 ± 1.51 ^ab^	41.50 ± 2.09 ^ab^	14.37 ± 1.26 ^c^	59.04 ± 1.47 ^d^	9.31 ± 0.51 ^e^	40.23 ± 0.80 ^b^	32.34 ± 2.76 ^a^	42.22 ± 0.67 ^ab^	**	0.0013
13	35.9	Coconut	Coconut	0.27 ± 0.03 ^a^	0.49 ± 0.10 ^bc^	0.50 ± 0.03 ^b^	0.37 ± 0.02 ^c^	0.50 ± 0.03 ^b^	0.08 ± 0.01 ^d^	0.32 ± 0.01 ^a^	0.58 ± 0.08 ^b^	0.70 ± 0.04 ^e^	0.46 ± 0.04 ^b^	**	0.0010
14	36.4	Fatty, coconut	Fatty, waxy	1.74 ± 0.20 ^a^	2.64 ± 0.33 ^b^	2.34 ± 0.07 ^b^	1.74 ± 0.07 ^a^	6.81 ± 0.63 ^c^	3.63 ± 0.08 ^d^	0.38 ± 0.04 ^e^	1.37 ± 0.15 ^a^	0.93 ± 0.14 ^a^	3.91 ± 0.25 ^d^	NS	0.7000

^a^ Different superscripts for each volatile compound within the same line indicate statistically significantly different values (*p* < 0.05). ^b^ Codes—1: 2-Heptanone; 2: Methoxyphenyl oxime; 3: Ethyl hexanoate; 4: Hexanoic acid; 5: Phenylacetaldehyde; 6: 2-Nonanone; 7: Ethyl octanoate; 8: Octanoic acid; 9: 2-Undecanone; 10: Nonanoic acid; 11: Ethyl decanoate; 12: Decanoic acid; 13: δ-Decalactone; 14: Dodecanoic acid. ^c^ Odor and flavor descriptions retrieved from http://www.thegoodscentscompany.com. Last access 03 September 2023 [43]. NS: not significant at *p* < 0.05. *, **, and ***, significant at *p* < 0.05, 0.01, and 0.001, respectively.

**Table 5:** Results of the parameters obtained in the texture, color, pH, water activity and humidity analyses. Mean ± Standard Deviation (n = 9). ***, significant at *p* < 0.001. Different superscripts within the same line indicate statistically significantly different values (*p* < 0.05).

	Force at Fracture (N)	Deformation at Fracture (%)	Work for Fracture (N.mm)	Maximum Force for Compression (N)	L	a*	b*	Aw	Humidity	pH
ANOVA	***	***	***	***	***	***	***	***	***	***
M1	28.25 ± 5.94 ^b^	41.63 ± 2.80 ^g^	72.37 ± 12.80 ^b^	30.50 ± 6.53 ^b^	65.66 ± 4.05 ^de^	−2.66 ± 0.49 ^bcd^	12.34 ± 1.73 ^f^	0.86 ± 0.00 ^c^	27.48 ± 1.30 ^de^	5.34 ± 0.08 ^d^
M2	27.06 ± 6.69 ^bc^	53.21 ± 5.96 ^de^	103.38 ± 29.94 ^a^	28.23 ± 6.84 ^bc^	67.23 ± 6.50 ^cd^	−2.20 ± 1.21 ^ab^	14.28 ± 2.20 ^cde^	0.87 ± 0.00 ^bc^	28.99 ± 2.44 ^cd^	5.53 ± 0.08 ^abc^
M3	20.99 ± 4.01 ^e^	54.03 ± 4.53 ^d^	61.79 ± 14.25 ^bcd^	21.15 ± 3.95 ^f^	68.23 ± 4.57 ^bcd^	−2.90 ± 0.94 ^cd^	14.22 ± 0.70 ^de^	0.86 ± 0.00 ^c^	25.70 ± 3.29 ^e^	5.43 ± 0.06 ^bcd^
M4	38.87 ± 13.01 ^a^	51.38 ± 4.78 ^e^	99.39 ± 38.72 ^a^	40.22 ± 10.74 ^a^	63.40 ± 4.74 ^e^	−3.14 ± 0.74 ^d^	13.80 ± 1.82 ^de^	0.85 ± 0.01 ^d^	28.48 ± 2.00 ^cde^	5.41 ± 0.13 ^cd^
M5	21.77 ± 5.03 ^de^	47.72 ± 1.70 ^f^	49.18 ± 12.90 ^e^	21.98 ± 4.92 ^ef^	76.04 ± 4.55 ^a^	−1.71 ± 0.80 ^a^	17.48 ± 2.04 ^a^	0.86 ± 0.01 ^bc^	26.82 ± 3.45 ^de^	5.14 ±0.10 ^e^
M6	24.80 ± 5.63 ^bcd^	51.00 ± 3.41 ^e^	59.63 ± 17.36 ^cde^	25.21 ± 5.06 ^cde^	67.75 ± 5.81 ^cd^	−2. 70 ± 1.12 ^bcd^	13.54 ± 1.36 ^e^	0.87 ± 0.01 ^bc^	31.04 ± 1.01 ^bc^	5.34 ± 0.12 ^d^
M7	20.35 ± 4.24 ^e^	62.52 ± 2.12 ^b^	74.00 ± 18.04 ^b^	20.65 ± 4.16 ^f^	70.82 ± 5.25 ^b^	−2.31 ± 0.86 ^b^	14.28 ± 1.32 ^cde^	0.87 ± 0.00 ^bc^	32.38 ± 0.89 ^b^	5.65 ± 0.10 ^a^
M8	12.61 ± 2.73 ^f^	66.39 ± 2.69 ^a^	50.64 ± 12.14 ^de^	12.75 ± 2.66 ^g^	78.76 ± 2.27 ^a^	−2.56 ± 0.26 ^bc^	15.28 ± 1.73 ^bc^	0.89 ± 0.01 ^a^	38.70 ± 0.59 ^a^	5.45 ± 0.18 ^bcd^
M9	23.28 ± 3.08 ^cde^	56.53 ± 1.92 ^c^	68.21 ± 10.19 ^bc^	23.40 ± 3.02 ^def^	65.58 ± 3.58 ^de^	−3.11 ± 1.00 ^d^	14.68 ± 1.23 ^cd^	0.87 ± 0.01 ^b^	28.50 ± 0.51 ^cde^	5.47 ± 0.44 ^bcd^
M10	26.16 ± 7.22 ^bc^	57.10 ± 5.93 ^c^	53.24 ± 20.98 ^de^	26.50 ± 7.27 ^cd^	69.64 ± 3.44 ^bc^	−2.91 ± 0.94 ^cd^	15.87 ± 2.06 ^b^	0.86 ± 0.00 ^cd^	28.89 ± 2.01 ^cd^	5.58 ± 0.10 ^ab^

**Table 6:** Consumer satisfaction degree of Spanish cured ewes’ cheeses ^τ^, compared between PDO and Non-PDO groups.

Attribute	ANOVA	M1	M2	M3	M4	M5	M6	M7	M8	M9	M10	ANOVA PDO/Non-PDO	*p*-Value
Overall liking	***	6.3 ± 2.1 ^ab^	6.3 ± 1.7 ^ab^	6.6 ± 1.5 ^a^	6.5 ± 1.8 ^a^	6.4 ± 1.8 ^ab^	6.1 ± 2.1 ^ab^	4.9 ± 0.5 ^b^	6.4 ± 1.7 ^ab^	5.9 ± 2.0 ^ab^	6.4 ± 2.1 ^ab^	***	0.0003
Color	***	6.6 ± 1.8 ^b^	6.7 ± 1.7 ^b^	7.1 ± 1.4 ^a^	6.4 ± 1.9 ^bc^	6.2 ± 1.8 ^c^	6.6 ± 1.4 ^ab^	6.8 ± 1.5 ^b^	6.6 ± 1.8 ^b^	6.0 ± 1.9 ^c^	6.5 ± 1.7 ^bc^	***	0.0000
Odor	NS	5.9 ± 1.9	6.1 ± 2.0	6.3 ± 1.7	6.1 ± 2.0	5.8 ± 2.2	6.0 ± 1.9	5.9 ± 1.8	5.8 ± 1.6	5.6 ± 1.7	6.1 ± 1.9	***	0.0003
Firmness	**	6.2 ± 1.7 ^ab^	5.4 ± 2.1 ^c^	6.4 ± 1.6 ^a^	6.1 ± 1.9 ^b^	6.3 ± 1.9 ^ab^	6.5 ± 1.6 ^a^	5.8 ± 2.1 ^b^	5.5 ± 2.0 ^bc^	6.2 ± 1.9 ^ab^	6.4 ± 1.6 ^a^	***	0.0002
Crumbliness	**	6.0 ± 1.9 ^ab^	5.6 ± 2.0 ^b^	6.0 ± 2.1 ^ab^	5.8 ± 1.9 ^b^	6.3 ± 1.9 ^a^	6.1 ± 1.8 ^ab^	5.8 ± 1.8 ^b^	5.6 ± 1.9 ^b^	5.7 ± 1.8 ^b^	6.2 ± 1.8 ^a^	***	0.0004
Sourness	NS	6.0 ± 1.9	5.8 ± 1.9	6.2 ± 1.6	6.0 ± 1.7	6.1 ± 1.9	5.3 ± 1.9	6.3 ± 1.9	5.7 ± 1.8	5.9 ± 1.8	5.6 ± 1.8	***	0.0004
Saltiness	NS	6.3 ± 1.8	6.3 ± 2.0	6.4 ± 1.6	6.6 ± 1.5	6.4 ± 1.8	5.9 ± 1.7	6.2 ± 1.7	6 ± 1.5	6.2 ± 1.6	6.0 ± 1.7	***	0.0001
Piquant	NS	5.8 ± 1.7	5.4 ± 2.0	5.7 ± 1.6	5.9 ± 1.5	6.0 ± 1.8	5.4 ± 1.8	5.6 ± 1.8	5.6 ± 1.7	5.8 ± 1.7	5.7 ± 1.8	***	0.0007
Ewe flavor	NS	6.2 ± 1.9	6 ± 1.8	6.2 ± 1.5	6.2 ± 1.7	6.4 ± 2.0	5.9 ± 2.1	5.7 ± 1.8	6.1 ± 1.6	5.9 ± 2.0	6.2 ± 2.0	***	0.0002
Aftertaste	***	6.2 ± 2.1 ^a^	5.9 ± 2.2 ^ab^	6.1 ± 2.0 ^ab^	6.4 ± 1.9 ^a^	5.8 ± 2.2 ^c^	5.1 ± 2.3 ^c^	6.3 ± 2.0 ^a^	6.2 ± 1.7 ^a^	6.1 ± 2.0 ^ab^	6 ± 2.3 ^ab^	***	0.0003

NS: not significant at *p* > 0.05; **, and *** were significant at *p* < 0.01, and 0.001, respectively. ^τ^ Different superscripts for each attribute within the same line indicate statistically significantly different values (*p* < 0.05).

## Data Availability

Data is contained within the article.
